# The EUTOS long‐term survival score predicts disease‐specific mortality and molecular responses among patients with chronic myeloid leukemia in a practice‐based cohort

**DOI:** 10.1002/cam4.3516

**Published:** 2020-10-10

**Authors:** Eriko Sato, Noriyoshi Iriyama, Michihide Tokuhira, Tomoiku Takaku, Maho Ishikawa, Tomonori Nakazato, Kei‐Ji Sugimoto, Hiroyuki Fujita, Yuta Kimura, Isao Fujioka, Norio Asou, Norio Komatsu, Masahiro Kizaki, Yoshihiro Hatta, Tatsuya Kawaguchi

**Affiliations:** ^1^ Department of Hematology Juntendo University Nerima Hospital Tokyo Japan; ^2^ Division of Hematology and Rheumatology Department of Medicine Nihon University School of Medicine Tokyo Japan; ^3^ Department of Hematology Saitama Medical Center Saitama Medical University Saitama Japan; ^4^ Department of Hematology Juntendo University School of Medicine Tokyo Japan; ^5^ Department of Hemato‐Oncology Saitama Medical University International Medical Center Saitama Japan; ^6^ Department of Hematology Yokohama Municipal Citizen's Hospital Yokohama Japan; ^7^ Department of Hematology Juntendo University Urayasu Hospital Urayasu Japan; ^8^ Department of Hematology Saiseikai Yokohama Nanbu Hospital Yokohama Japan; ^9^ Department of Hematology and Infectious Diseases Kumamoto University Hospital Kumamoto Japan; ^10^ Department of Medical Technology Kumamoto Health Science University Kumamoto Japan

**Keywords:** chronic myeloid leukemia, ELTS score, EUTOS, prognosis, tyrosine kinase inhibitor

## Abstract

The European Treatment and Outcome Study (EUTOS) long‐term survival (ELTS) score predicts disease‐specific death in patients with chronic myeloid leukemia (CML) being treated with imatinib during the chronic phase (CP) of the disease. However, it is unclear whether the ELTS score predicts CML‐related events or treatment responses. This study evaluated the predictive value of the ELTS score regarding prognosis and treatment response in patients with CML‐CP. Clinical data were retrospectively obtained from patients enrolled in the CML Cooperative Study Group (CML‐CSG), which included patients diagnosed with CML‐CP from April 2001 to January 2016, and treated with any tyrosine kinase inhibitor (TKI) as first‐line therapy. Among 342 eligible patients, the ELTS scores indicated low‐, intermediate‐, and high‐risk in 74%, 21%, and 5% of patients, respectively. Patients with high ELTS scores had significantly higher disease‐specific mortality and worse event‐free survival, progression‐free survival, and overall survival. Among four risk scores, including the Sokal, Hasford, EUTOS, and ELTS scores, risk stratification by the ELTS score had the highest predictive value in assessing patient prognosis, and also in treatment responses. In fact, the EUTOS and ELTS scores were able to predict the major molecular response within 12 months. Most importantly, the ELTS score was the only scoring system that predicted deep molecular response at any time, regardless of risk level (65.0%, 43.7%, and 23.5% in low‐, intermediate‐, and high‐risk groups, respectively). Compared to other risk scores, the ELTS score was the most sensitive risk classification tool for the four endpoints of interest in this study, as well as molecular responses in patients with CML‐CP.

## Graphical abstract

In this real‐world TKIs treated CML‐CP patients cohort, the EUTOS long‐term survival (ELTS) score was the most sensitive risk classification tool to predict CML‐specific deaths, EFS, PFS, and OS compared to the Sokal, Hasford, and EUTOS scoring systems. Notably, the ELTS score was the only scoring system that predicted cumulative incidence of deep molecular response.

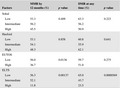

## 1 INTRODUCTION

After the introduction of the ABL tyrosine kinase inhibitor (TKI), imatinib, prognosis associated with chronic myeloid leukemia (CML) significantly improved. In fact, according to recent reports, the life expectancy of CML patients is comparable to that of the general population.^1^, ^2^ Moreover, the development and certification of second‐generation TKIs (2G‐TKIs) such as nilotinib and dasatinib, with greater potency and more tolerable adverse effects, as first‐line therapy for the chronic phase of CML (CML‐CP) have further increased disease control rates. As a result, nowadays, fewer patients and clinicians worry about death “due to leukemia” than about “death due to reasons other than leukemia.” Given these developments, it is crucial to have a predictive tool to precisely identify CML‐CP patients with a high risk of CML‐related death.

Before the TKI era, two prognostic tools were predominantly used for assessing risk in CML‐CP patients; the Sokal and Hasford scores. These scores were established in 1984 and 1998, during the conventional chemotherapy and splenectomy era, and the interferon era, respectively. After the introduction of imatinib, the European Treatment and Outcome Study (EUTOS) score, published in 2011, was developed to predict a complete cytogenetic response (CCyR) at 18 months after treatment initiation, which was used as a surrogate marker for survival.^3^, ^4^, ^5^ A previous study has reported that the EUTOS score is the best currently available scoring system for predicting event‐free survival (EFS), progression‐free survival (PFS), and CML‐associated mortality among patients with CML‐CP receiving imatinib.^6^ Nevertheless, with the introduction of 2G‐TKIs, the EUTOS score may be unreliable in patients who receive these new treatments. We have previously reported that treatment with 2G‐TKIs might reduce the risk of disease progression, as defined by the EUTOS score.^7^ As such, the most predictable scoring system for current treatment trends has room for consideration.

The EUTOS long‐term survival (ELTS) score was established in 2016 for the identification and prediction of potent “leukemic death” in patients treated with imatinib.^8^ Although the use of TKIs means that most patients with CML‐CP do not die due to their primary disease, some patients are resistant to TKIs and, therefore, require early alternative therapeutic interventions.

In April 2009, 2G‐TKIs were approved for use in imatinib‐resistant or intolerant patients. Subsequently, after April 2011, 2G‐TKIs were approved as initial therapy agents for CML‐CP patients in Japan. Therefore, we wonder whether the ELTS score can accurately predict CML‐related death, EFS, PFS, and OS, as well as the achievement rates of major molecular response (MMR) and deep molecular response (DMR) in CML‐CP patients. This study aimed to validate the use of the ELTS score by comparing it with other prognostic scores in a cohort of patients recruited in a real‐world setting.

## 2 METHODS

### 2.1 Patients

Data from patients registered in the CML Cooperative Study Group (CML‐CSG), which is a collaboration of four university hospitals and five university branch hospitals, were retrospectively reviewed. Patients were eligible for inclusion if they were aged 15 years or older, diagnosed with CML‐CP from April 2001 to January 2016, and treated with any TKI as initial therapy. Since April 2011, 2G‐TKIs have been available as initial therapy for CML‐CP patients in Japan. The study protocol was approved by the research ethics board of each participating institution and conducted in accordance with the principles of the Declaration of Helsinki.

### 2.2 Prognosis and response assessments

In the present study, disease progression was defined as the transformation to the accelerated phase/blastic phase (AP/BP) of the disease. Incidence of any event included loss of treatment efficacy, including loss of complete hematologic response (CHR) after CHR had previously been achieved, loss of partial cytogenetic response (PCyR) after PCyR had previously been achieved, or loss of CCyR after CCyR had previously been achieved, disease progression, and death due to any cause. The molecular response was evaluated by measuring the *BCR*‐*ABL1* transcript using real‐time quantitative PCR or a transcription‐mediated amplification and hybridization protection (TMA) assay. *BCR*‐*ABL1* transcript levels ≤0.1% according to the International Scale (IS), or ≤100 copies/μg RNA as determined using the TMA assay, were considered an MMR, while transcript levels ≤0.0032% were considered a DMR as described previously.^9^

### 2.3 Statistical analyses

For every patient, PFS was defined as the time from initial treatment with TKIs to confirmed disease progression or the last follow‐up consultation. EFS was defined as the time from initial treatment with TKIs to the first occurrence of any of the events of interest or the last follow‐up consultation. The OS was defined as the time from initial treatment with TKIs to death due to any cause or the last follow‐up consultation. In the analysis of CML‐specific mortality, deaths unrelated to CML were censored at the time of demise. The Kaplan‐Meier method and the log‐rank test were utilized to estimate and compare the PFS, EFS, OS, and CML‐specific mortality rates. Fisher's exact test was utilized to determine statistical significance in each group. Statistical analyses were performed using EZR (Saitama Medical Center, Jichi Medical University), a graphical user interface for the R programming language (The R Foundation for Statistical Computing, Vienna, Austria).^10^

## 3 RESULTS

### 3.1 Patient characteristics

There were 342 eligible patients newly diagnosed with CML‐CP during the study period. Patient characteristics are shown in Table 1. A total of 150 (44%), 137 (40%), and 55 (16%) patients were classified as low‐, intermediate‐, and high‐risk according to the Sokal score; 143 (42%), 170 (50%), and 29 (8%) as low‐, intermediate‐, and high‐risk according to the Hasford score; 293 (86%) and 49 (14%) as low‐ and high‐risk according to the EUTOS score; and 254 (74%), 71 (21%), and 17 (5%) as low‐, intermediate‐, and high‐risk according to the ELTS score, respectively. Regarding first‐line therapy, 172 (50.3%) patients were treated with imatinib and 170 (49.7%) with a 2G‐TKI (specifically, 93 with dasatinib and 77 with nilotinib).

**Table 1:** Baseline characteristics of the patients at the time of treatment initiation

Factors	*n* = 342
Age (median, range)	53.5 (18–89)
Sex male ‐ no. (%)	212 (62)
Sokal ‐ no. (%)	
Low	150 (44)
Intermediate	137 (40)
High	55 (16)
Hasford ‐ no. (%)	
Low	143 (42)
Intermediate	170 (50)
High	29 (8)
EUTOS ‐ no. (%)	
Low	293 (86)
High	49 (14)
ELTS ‐ no. (%)	
Low	254 (74)
Intermediate	71 (21)
High	17 (5)
Initial therapy ‐ no. (%)	
Imatinib	172 (50)
Dasatinib	93 (27)
Nilotinib	77 (23)

Abbreviations: ELTS, EUTOS long‐term survival; EUTOS, European Treatment and Outcome Study.

### 3.2 CML‐related death

**Figure 1:**
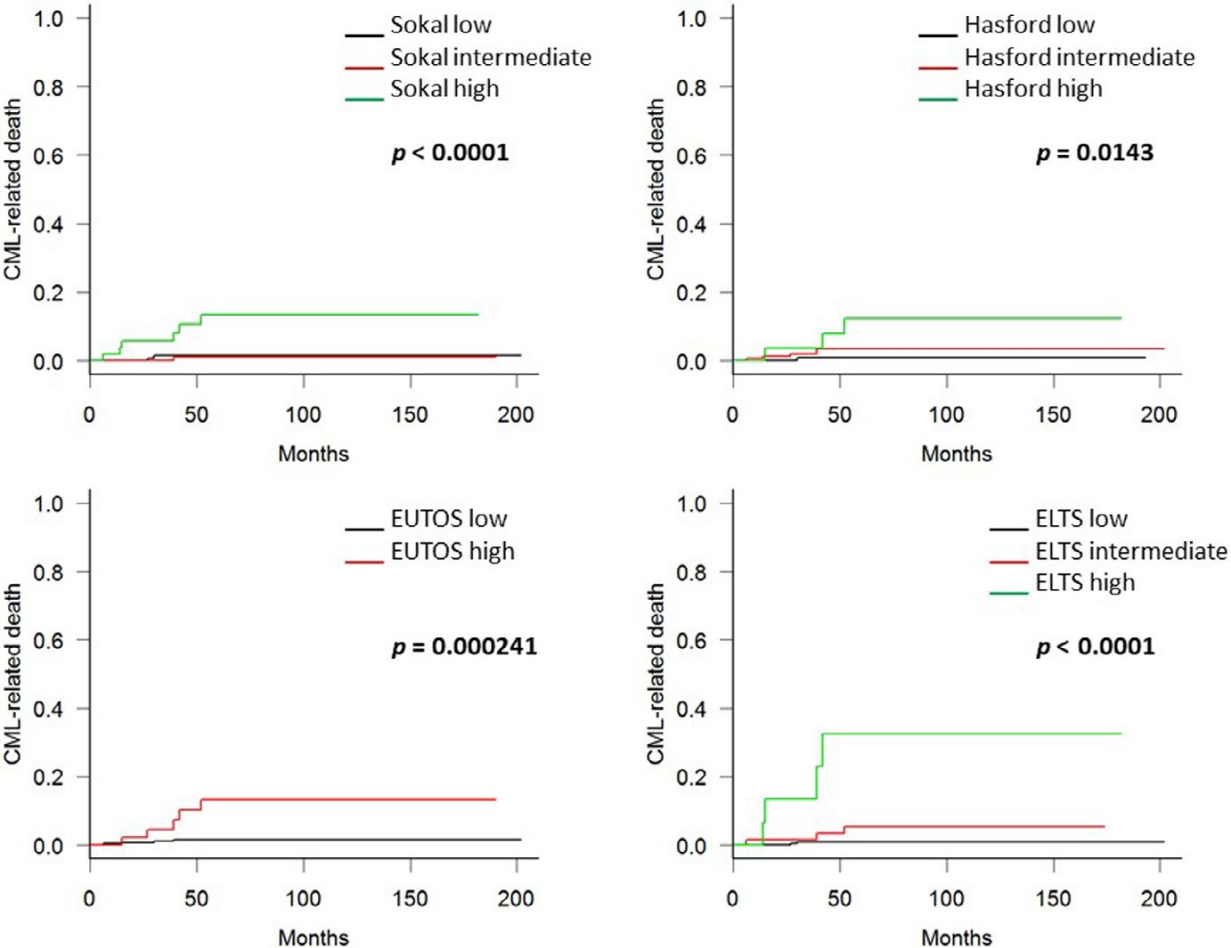
Kaplan‐Meier curves for chronic myeloid leukemia (CML)‐related deaths in this study cohort. Patients’ prognoses were made according to the Sokal, Hasford score, European Treatment and Outcome Study (EUTOS), and EUTOS long‐term survival (ELTS) score. The 5‐year CML‐related death rates according to the ELTS scores were 0.9%, 5.4%, and 32.6% for low‐, intermediate‐, and high‐risk patients, respectively. Patients defined as high‐risk according to the ELTS score had significantly worse CML‐related deaths outcomes than patients defined as intermediate‐ or low‐risk. The Sokal and Hasford scores were predictive of CML‐related deaths, but their sensitivity was lower compared to the ELTS score

Cumulative incidence rates of 5‐year CML‐related death, stratified by scoring systems, were as follows. The Sokal score (1.4% in low‐, 1.1% in intermediate‐, and 13.4% in high‐risk groups; *p* < 0.0001), Hasford score (0.8% in low‐, 3.5% in intermediate‐, and 12.4% in high‐risk groups; *p* = 0.0143), EUTOS score (1.5% in low‐ and 13.1% in high‐risk groups; *p* = 0.000241), and ELTS score (0.9% in low‐, 5.4% in intermediate‐, and 32.6% in high‐risk groups; *p* < 0.0001). Therefore, the ELTS score was the most sensitive predictor of CML‐related death in this study (Figure 1).

### 3.3 Prognostic value of the scoring systems in predicting EFS, PFS, and OS

**Figure 2:**
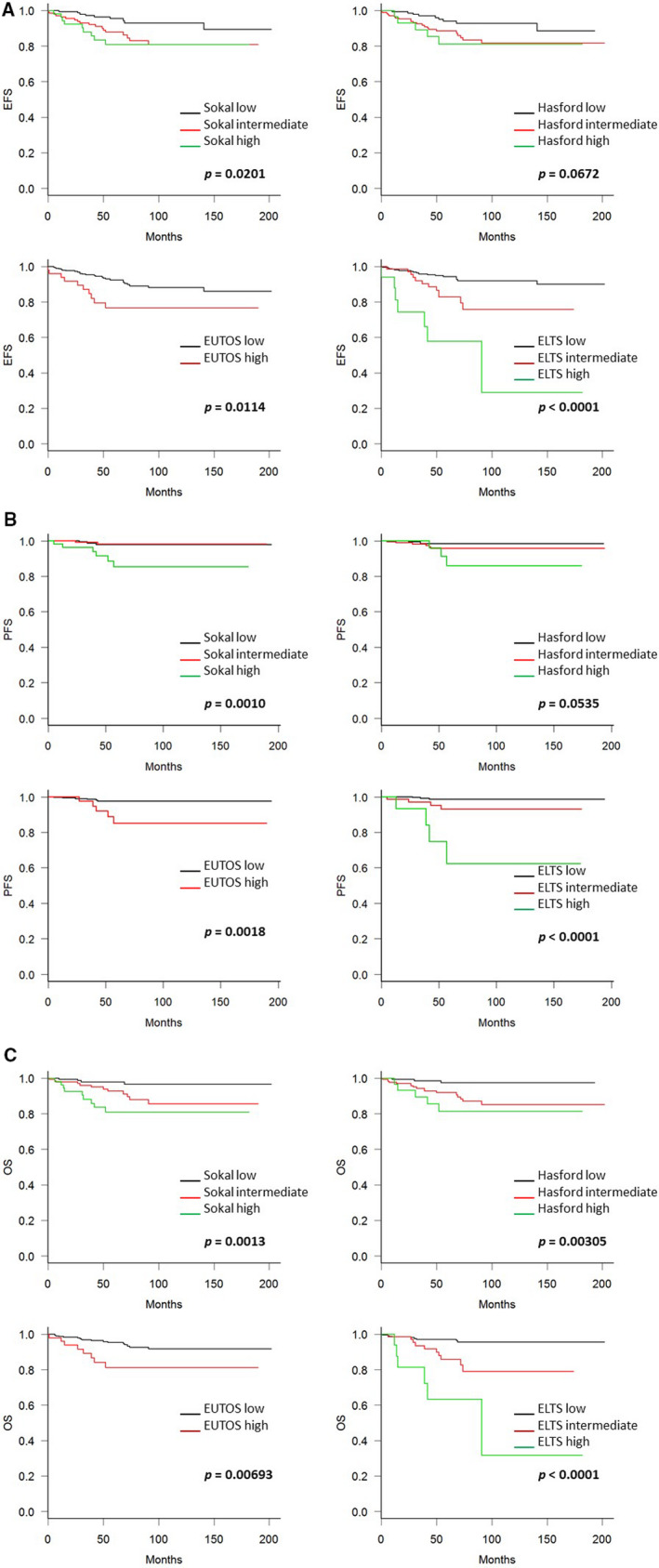
Kaplan‐Meier curves for (A) event‐free survival (EFS), (B) progression‐free survival (PFS), and (C) overall survival (OS). Hasford score could predict statistical difference in PFS and OS except EFS, Sokal, and Hasford scores could predictive of EFS, PFS, and OS, but again less sensitive than European Treatment and Outcome Study (EUTOS) long‐term survival (ELTS) score

Next, we validated the predictive ability of the four scoring systems for EFS, PFS, and OS. Figure 2A‐C (A: EFS, B: PFS, and C: OS) show the patient survival rates defined by each scoring system. High‐risk patients, as defined by the ELTS score, had worse EFS, PFS, and OS compared to patients defined as low‐ or intermediate‐risk. The Sokal and EUTOS scores were also predictive of EFS and PFS, but the Hasford score failed. Although the Sokal, Hasford, and EUTOS scores could predict OS, risk stratification by the ELTS score appeared to be the most reliable of the tested scores at predicting patients’ prognoses.

### 3.4 Molecular responses

Then, we assessed molecular responses stratified by these scoring systems. As shown in Table 2, patients defined as high‐risk by the ELTS score had a significantly lower MMR attainment than did intermediate‐ and low‐risk patients; the 12‐month MMR attainment rates were 56.3% in low‐, 52.1% in intermediate‐, and 11.8% in high‐risk groups (*p* = 0.00137). The EUTOS score could also predict MMR attainment at 12 months (56.0% in low‐ and 36.7% in high‐risk groups; *p* = 0.0136), whereas the Sokal and Hasford scores could not predict this outcome.

**Table 2:** Response to tyrosine kinase inhibitor therapy stratified by scoring systems

Factors	MMR by 12 months (%)	*p* value	DMR at any time (%)	*p* value
Sokal				
Low	53.3	0.409	63.3	0.223
Intermediate	56.2		56.2	
High	45.5		50.9	
Hasford				
Low	53.1	0.858	60.8	0.641
Intermediate	54.1		55.9	
High	48.3		62.1	
EUTOS				
Low	56.0	0.0136	59.7	0.275
High	36.7		51.0	
ELTS				
Low	56.3	0.00137	65.0	0.0000569
Intermediate	52.1		43.7	
High	11.8		23.5	

Abbreviations: DMR, deep molecular response; ELTS, EUTOS long‐term survival; EUTOS, European Treatment and Outcome Study; MMR, major molecular response

Most importantly, the ELTS score was the only scoring system able to predict the attainment of DMR at any time; 65.0% in low‐, 43.7% in intermediate‐, and 23.5% in high‐risk groups; *p* = 0.0000569. The Sokal, Hasford, and EUTOS scores were not able to differentiate the attainment of DMR.

Furthermore, we compared the concordance and discordance of MMR attainment rates at 12 months among the risk groups between the ELTS and other scoring systems. Table 3 shows that, for patients defined as low‐ and intermediate‐risk by the ELTS score, the MMR rates were between 37.5 and 60.0%, even if these patients were classified as high‐risk by other scoring systems. On the contrary, for patients who were classified as high‐risk by the ELTS score, the MMR rates were much lower (0‐25.0%).

**Table 3:** Major molecular response rates by 12 months concerning a discordance between scoring systems

ELTS	Low *n* (%)	Intermediate *n* (%)	High *n* (%)
Sokal			
Low, *n* (%)	78/145 (53.8)	2/5 (40)	0/0
Intermediate, *n* (%)	50/84 (59.5)	27/47 (57.4)	0/6 (0)
High, *n* (%)	15/25 (60)	8/19 (42.1)	2/11 (18.2)
Hasford			
Low, *n* (%)	73/134 (54.5)	3/8 (37.5)	0/1 (0)
Intermediate, *n* (%)	63/108 (58.3)	29/54 (53.7)	0/8 (0)
High, *n* (%)	7/12 (58.3)	5/9 (55.6)	2/8 (25)
EUTOS			
Low, *n* (%)	132/228 (57.9)	32/58 (55.2)	0/7 (0)
High, *n* (%)	11/26 (42.3)	5/13 (38.5)	2/10 (20)

Abbreviations: EUTOS, European Treatment and Outcome Study; ELTS, EUTOS long‐term survival.

### 3.5 Causes of disease progression

In this study, an event of interest occurred in 36 patients, including 16 who died of a cause unrelated to CML, and 11 who progressed to AP/BP. Among patients who progressed to AP/BP, nine subsequently died of leukemia. As shown in Table 4, patients defined as high‐risk by the ELTS score particularly had a higher frequency of disease progression (4/17), although disease progression was seen in some low‐ and intermediate‐risk patients also (3/254 in low and 4/71 in intermediate‐risk groups). The numbers of patients who died of leukemia were 2/254 (0.79%), 3/71 (4.22%), and 4/17 (23.5%) in low‐, intermediate‐, and high‐risk patients by the ELTS score, respectively. Furthermore, low‐risk patients by the ELTS score were unlikely to progress to AP/BP, regardless of classification as high‐risk according to other scoring systems.

### 3.6 CML‐related death rates according to the choice of initial therapy

**Figure 3:**
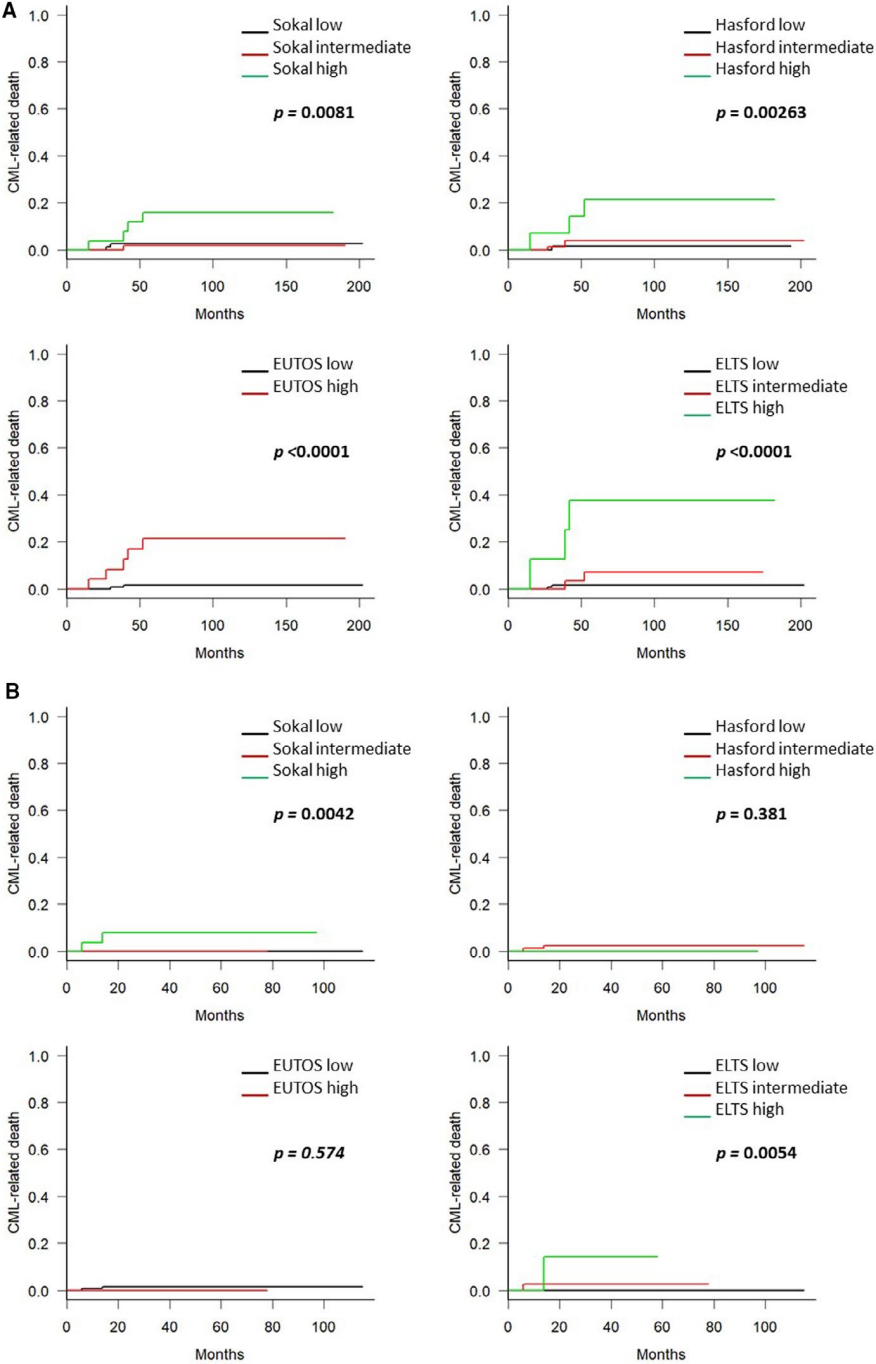
Cumulative incidence of chronic myeloid leukemia (CML)‐related deaths stratified by the scoring systems, according to initial therapy. The prognoses of patients initially treated with imatinib (A) and with second‐generation tyrosine kinase inhibitor (B) were stratified by the Sokal, Hasford, European Treatment and Outcome Study (EUTOS), and EUTOS long‐term survival (ELTS) score, respectively

Finally, we investigated whether the choice of first‐line therapy affected the predictability of the risk stratification for CML‐related deaths according to initial therapy. As shown in Figure 3, all scoring systems could predict the CML‐related death in the imatinib‐treated group (Figure 3A); however, this could be predicted only by the Sokal and ELTS scoring systems in the 2G‐TKI‐treated group (Figure 3B).

## 4 DISCUSSION

Several factors influence the survival of CML‐CP patients, including clonal chromosomal abnormalities (CCA) in Philadelphia chromosome‐positive cells, BCR‐ABL1 kinase domain mutations, non‐ABL kinase dependencies, and multidrug resistance polymorphisms. However, these examinations, excluding CCA, are not commonly available in clinical practice.^11^, ^12^, ^13^, ^14^, ^15^ As such, four evaluation scores have been developed; the Sokal and Hasford scores were developed before the introduction of the TKIs, while the EUTOS and ELTS scores were developed afterward. This study aimed to assess the ability of the ELTS score in predicting disease‐specific mortality, patient survival, and molecular responses in comparison with the other three scoring systems, using a real‐world cohort of CML‐CP patients who had been started on treatment with all available first‐line TKIs.

In the present study, the ELTS score accurately predicted survival indicators of interest, specifically, CML‐specific mortality, EFS, PFS, and OS. In this study cohort, 11 out of 349 patients progressed to AP/BP; among them, nine died of leukemia. Among patients in the low‐, intermediate‐, and high‐risk groups based on the ELTS score, 2/254 (0.79%), 3/71 (4.22%), and 4/17 (23.5%) died of leukemia, respectively. Furthermore, low‐risk patients based on the ELTS score were unlikely to progress to AP/BP, regardless of whether they were classified as high‐risk according to other scoring systems. Therefore, these findings suggest that ELTS can accurately assess the risk of disease‐specific mortality among high‐risk patients. A previous study conducted with 339 imatinib‐treated patients and 78 2G‐TKIs‐treated patients validated the capability of the ELTS score compared with other scores, and the ELTS score allows for a more precise stratification of the risk of CML‐related death.^16^ Another recent study from China, which investigated patients treated with imatinib as first‐line therapy, presented the prognostic value of the ELTS score compared with the other three risk scores. ELTS score could predict outcomes of CCyR, PFS, OS, and it was the only scoring system that could predict CML‐related death.^17^ Our study also provided positive evidence supporting the ELTS score as the most valuable risk stratification tool for CML‐CP patients. Even if limited to the 2G‐TKIs treated patients, the ELTS score could stratify CML‐related deaths among patients classified into each risk group. However, the EUTOS score could not discriminate between high‐ and low‐risk patients, according to our previous study.^7^

Next, we investigated whether the ELTS score could anticipate molecular response rates; specifically, MMR at 12 months and DMR at any time after treatment initiation. Among patients defined as low‐ and intermediate‐risk by the ELTS score, the MMR rates at 12 months were 37.5‐60.0%, even if the patients were classified as high risk by other scoring systems. However, the MMR rates were much lower (0‐25.0%) for patients who were classified as high‐risk by the ELTS score. The ELTS score was able to predict the MMR rate, which is considered a surrogate measure of the risk of CML‐specific death, while other risk assessment tools could not. This finding was consistent with findings from a previous study, which analyzed 709 patients receiving imatinib and 244 patients receiving 2G‐TKI as first‐line treatment.^18^ To the best of our knowledge, the present study is the first study to report the value of the ELTS score in predicting the cumulative incidence of DMR at any time during treatment. The ELTS accurately extracted high‐risk patients unlikely to attain DMR with a significant statistical difference (*p* = 0.0000569). With this scoring system, not only could we infer the high possibility of CML‐related death in high‐risk patients, but we could also deduce treatment‐free capable patients who had been treated with TKIs and are in remission from the low‐risk group.

The limitations of our study include its retrospective nature, the small sample size, and the unavailability of treatment compliance details. Validation in prospective studies would be needed to further refine the prognostic scoring systems. Another limitation is a possible selection bias since all patients were not analyzed, as the scoring for risk stratification was unavailable in 18 patients with CML‐CP enrolled in the database.

In conclusion, the findings from the present study support the predictive value of the ELTS score for CML‐CP patients who were started on treatment with all available first‐line TKIs. Our results showed the ELTS score most accurately predicts CML‐specific deaths and molecular responses in a real‐world clinical setting. Patients identified by the ELTS score as high‐risk might benefit from treatment with 2G‐TKI to faster achieve DMR. Our study provided positive evidence to support that the ELTS score is a better risk stratification tool for CML‐CP patients, and it aids clinicians to make therapeutic decisions on the appropriate first‐line treatment.

## 5 CONFLICT OF INTEREST

N Iriyama received honoraria and speaker fees from Bristol‐Myers Squibb, Novartis Pharma K.K., Otsuka Pharmaceutical, and Pfizer Inc. M Tokuhira received honoraria and speaker fees from Pfizer Inc. and Bristol‐Myers Squibb. M Kizaki received honoraria and speaker fees from Bristol‐Myers Squibb and Novartis Pharma K.K. T Takaku and Y Hatta received honoraria from Bristol‐Myers Squibb and Novartis Pharma K.K. T Kawaguchi received honoraria and speaker fees from Bristol‐Myers Squibb, Novartis Pharma K.K., and Pfizer Inc. The remaining authors declare no competing financial interests.

6

**Table 4:** Disassociations between ELTS score and other scoring systems (denominators of fractions), and distribution of patients who experienced disease progression (numerators of fractions)

ELTS	Low *n* = 254	Intermediate *n* = 71	High *n* = 17
Sokal			
Low, *n* = 150	3/145	0/5	0/0
Intermediate, *n* = 137	0/84	2/47	0/6
High, *n* = 55	0/25	2/19	4/11
Hasford			
Low, *n* = 143	1/134	1/8	0/1
Intermediate, *n* = 170	2/108	2/54	2/8
High, *n* = 29	0/12	1/9	2/8
EUTOS			
Low, *n* = 293	2/228	3/58	1/7
High, *n* = 49	1/26	1/13	3/10

Abbreviations: EUTOS, European Treatment and Outcome Study; ELTS, EUTOS long‐term survival.

## Data Availability

The data sets generated and/or analyzed during the current study are not publicly available to ensure patient privacy, but are available from the corresponding author on reasonable request.
